# Short-term markers of DNA damage among roofers who work with hot asphalt

**DOI:** 10.1186/s12940-016-0182-4

**Published:** 2016-10-20

**Authors:** Berrin Serdar, Stephen Brindley, Greg Dooley, John Volckens, Elizabeth Juarez-colunga, Ryan Gan

**Affiliations:** 1 Department of Environmental and Occupational Health, Colorado School of Public Health, University of Colorado Denver, Denver, USA; 2 Department of Epidemiology, Colorado School of Public Health, University of Colorado Denver, Denver, USA; 3 Analytical Toxicology Laboratory, Center for Environmental Medicine, Colorado State University, Fort Collins, USA; 4 Department of Mechanical Engineering, Colorado State University, Fort Collins, USA; 5 Department of Biostatistics and Informatics, Colorado School of Public Health, University of Colorado Denver, Denver, USA; 6 Colorado School of Public Health, University of Colorado Anschutz Medical Campus, Mail Stop B119-V20, 12850 East Montview Boulevard, Rm. V20-3126, Aurora, CO 80045 USA

**Keywords:** Polycyclic aromatic hydrocarbons, PAHs, Biomarkers, 8-hydroxy-deoxyguanosine, γH2AX, DNA double strand breaks

## Abstract

**Background:**

Roofers are at increased risk for various malignancies and their occupational exposures to polycyclic aromatic hydrocarbons (PAHs) have been considered as important risk factors. The overall goal of this project was to investigate the usefulness of phosphorylated histone H2AX (γH2AX) as a short-term biomarker of DNA damage among roofers.

**Methods:**

Blood, urine, and dermal wipe samples were collected from 20 roofers who work with hot asphalt before and after 6 h of work on Monday and Thursday of the same week (4 sampling periods). Particle-bound and gas-phase PAHs were collected using personal monitors during work hours. γH2AX was quantified in peripheral lymphocytes using flow cytometry and 8-hydroxy-2-deoxyguanosine (8-OHdG) was assessed in urine using ELISA. General linear mixed models were used to evaluate associations between DNA damage and possible predictors (such as sampling period, exposure levels, work- and life-style factors). Differences in mean biomarker and DNA damage levels were tested via ANOVA contrasts.

**Results:**

Exposure measurements did not show an association with any of the urinary biomarkers or the measures of DNA damage. Naphthalene was the most abundant PAH in gas-phase, while benzo(e)pyrene was the most abundant particle-bound PAH. Post-shift levels of γH2AX and 8-OHdG were higher on both study days, when compared to pre-shift levels. Cigarette smoking was a predictor of γH2AX and urinary creatinine was a predictor of urinary 8-OHdG. Between-subject variance to total variance ratio was 35.3 % for γH2ax and 4.8 % for 8-OHdG.

**Conclusion:**

γH2AX is a promising biomarker of DNA damage in occupational epidemiology studies. It has a lower within-subject variation than urinary 8-OHdG and can easily be detected in large scale groups. Future studies that explore the kinetics of H2AX phosphorylation in relation to chemical exposures may reveal the transient and persistent nature of this sensitive biomarker of early DNA damage.

**Electronic supplementary material:**

The online version of this article (doi:10.1186/s12940-016-0182-4) contains supplementary material, which is available to authorized users.

## Background

Workers around the world experience daily exposures to potentially carcinogenic chemicals. Identifying the role of these exposures in cancer development later in life has been a major challenge in occupational epidemiology. Estimating the exposure-cancer association becomes more complicated by simultaneous exposures to other environmental and lifestyle factors. A further challenge is the long latency period between carcinogenic exposure and cancer diagnosis. Many times occupational studies rely on estimates of current exposures and their association to short-term markers of health effects. Among these markers, measures of DNA damage are viewed as reliable indicators of increased cancer risk [1], as they represent early signs of endogenous genomic instability at the tissue level and can help identify precancerous lesions which in turn can improve prevention efforts [2].

Roofers are at increased risk for different malignancies such as lung, bladder, stomach, skin and buccal cavity cancers, and leukemia [3–10]. Exposure to asbestos, polycyclic aromatic hydrocarbons (PAHs) and high rates of cigarette smoking have all been considered possible crucial risk factors among roofers [8, 11]. Work with hot asphalt is an important source of PAH exposure in this group and has been linked to DNA strand breaks, DNA adducts and sister chromatid exchanges [10, 12–14]. Asphalt is a mixture of hundreds of different chemical compounds, containing some known human carcinogens such as benzo(a)pyrene (BaP), which can be absorbed through inhalation, dermal contact, or ingestion [15]. Since many PAHs in asphalt are ubiquitous in the environment, distinguishing occupational exposures from environmental (non-occupational) exposures is difficult. PAHs in air occur both in gaseous and particulate phase: smaller molecular weight PAHs, such as the 2-ring naphthalene, are found predominantly in the gas phase whereas higher molecular weight compounds, such as the 4-ring pyrene, are found mainly in the particulate phase [16]. Accurate measurement of individual PAHs in air has been difficult due to the complexity of the mixtures and the sensitivity of some individual PAHs to environmental or analytical conditions [16, 17]. Additionally, dermal contact can be a significant route of exposure in many work environments, including roofing [18–21]. Because measures of external PAH exposure have limitations, biological monitoring of PAHs has also been used for risk estimation [22–24]. However, studies monitoring exposures to high molecular weight PAHs have similar challenges, such as low levels of exposures, undetectable levels of biomarkers, complicated analytical techniques with low sensitivity, and weak correlations between exposure and biomarker levels [24–27]. We and others have previously proposed that urinary metabolites of the more volatile and abundant PAHs, such as naphthalene, could theoretically increase the sensitivity of the analytical procedures [27–29].

Exposure to PAHs can increase reactive oxygen species (ROS) formation in the body. When the cellular antioxidant defense system is disturbed, the increased amount of ROS can cause oxidative damage to biomolecules such as DNA, proteins and lipids. Recent studies have supported the link between insufficient cellular defense towards oxidative DNA damage and increased susceptibility to cancer development [30–33]. Urinary 8-hydroxy-2′-deoxyguanosine (8-OHdG), a byproduct of DNA repair and oxidative damage, is a reliable biomarker of general oxidative stress and DNA damage related to occupational and environmental exposures [31, 34, 35]. Very few studies have quantified occupational PAH exposures and concurrent DNA damage. Among these, studies that examined asphalt exposure have mainly looked at highway maintenance workers [10, 12–14, 36, 37]. We have previously assessed 8-OHdG in roofers who work with hot asphalt and observed a good correlation with this marker and 1-OHPyr at the end of the work-shift [38]. However, a major limitation of 8-OHdG is that many individual (metabolic events, life style factors such as smoking and alcohol consumption) and environmental (e.g., UV radiation) factors can cause levels of 8-OHdG to fluctuate [31, 35, 39].

Double-strand DNA breaks have also been linked to PAH exposures; this form of DNA damage can be measured using phosphorylated histone H2AX (γH2AX) in individual cells [40]. Increased levels of cellular γH2AX have been associated with exposure to radiation [41–43], cigarette smoke [44, 45], particulate matter [46], and other toxic agents [45, 47, 48]. In fact, γH2AX is considered to be a sensitive marker of DNA damage and increased cancer risk [49, 50]. One limitation for using γH2AX in population studies has been the labor-intensive analytic techniques. Immunofluorescence microscopy is the most commonly used method for detecting γH2AX, although flow cytometry, Western blotting and ELISA have also been used [51]. Both microscopy and cytometry-based methods have been suitable to evaluate γH2AX formation. The image cytometry and Laser Scanning Microscopy (LSC) methods have an advantage over flow cytometry because they enable counting and sizing of γH2AX foci, but they are also expensive and time consuming [52, 53]. A study recently proposed the use of blood smears prepared from a drop of blood, which may provide a feasible method of immunostaining in large scale studies [53].

Before a biomarker can be comfortably used in epidemiology studies, it needs to be validated based on the following criteria: 1) The relationship between the biomarker and exposure in question, 2) The formation, distribution and elimination of the biomarker in humans, 3) Variation of the biomarker between- and within- study participants, 4) Baseline values of the biomarker in the general population, and finally 5) Cost and difficulty of analytical techniques [54]. Here, we used flow cytometry to quantify γH2AX from peripheral blood lymphocytes of roofers who work with hot asphalt. The overall goal of this project was to determine the usefulness of γH2AX as a short-term marker of DNA damage in roofers exposed to PAHs in comparison to the widely used urinary 8-OHdG.

## Methods

### Study population and sample collection

Twenty roofers employed by one roofing company were recruited. The study site was a roof replacement project located in Colorado Springs, Colorado and was visited by the field study team over four weeks between July and September of 2013. Potential participants were informed about the study at the site and those who signed the informed consent under University of Colorado’s IRB (COMIRB) approved protocol (COMIRB Protocol # 12–0443) were recruited. Each week, a new group of workers participated in the study over two workdays: Monday and Thursday. Study questionnaires were administered before and after the work-shift in either English or Spanish; the latter applied by a Spanish speaking interviewer. Biological samples (urine and blood) were collected before and after 6 h of work. Study participants also provided hand wipes (with 3 ml sun flower oil) at each sampling period. Information on personal characteristics (age, height, weight, etc.), life-style factors (smoking, dietary PAH exposures, alcohol consumption), use of protective equipment during the study day (such as gloves, masks, etc.) and specific work tasks performed during the day (removing old roof, applying new roof) were collected via questionnaires. The before-work questionnaire focused on non-occupational sources of PAH exposures and the after-work questionnaires contained more detailed questions on work practices. Both of these questionnaires are provided in Additional file 1.

After the completion of morning questionnaires and collection of biological samples, the participants were given lightweight vests with air monitors to collect air samples from within each worker’s breathing zone air during the shift. The participants were then asked to return to their work. After 6 h of work participants returned the vests with the air monitors. The total number of samples collected from 20 participants is as follows: 79 urine, 79 blood, 40 air, and 79 dermal wipes. One of the participants had to leave the site on the second study day due to a family emergency and could not provide samples that afternoon (period 4).

### Air PAHs

Polycyclic aromatic hydrocarbons (PAHs) in ambient air were measured within the breathing zone of workers via personal sampling. Particle-bound PAHs, 4-ring and above, were collected using personal sampling pumps (SKC XR5000) fitted with PM_2.5_ sampling inlets (model 2.05, Mesa Labs, Inc.) and 37 mm Teflon filters. Gas-phase PAHs were collected immediately downstream of the filters using standard adsorbent tubes (XAD-2, 2 section, 75/150 mg sorbent). Method details are provided in Additional file 2.

### PAH metabolites and creatinine in urine

PAH metabolites were analyzed using an automated solid-phase extraction based on a method developed by Romanoff [55]. Details for the analysis of metabolites and urine creatinine are presented in Additional file 2.

### PAH levels on dermal wipes

Dermal exposure samples were collected using a previously published hand washing method with sunflower oil [56]. Details are presented in Additional file 2.

### Lymphocyte γH2AX flow cytometry

**Fig. 1:**
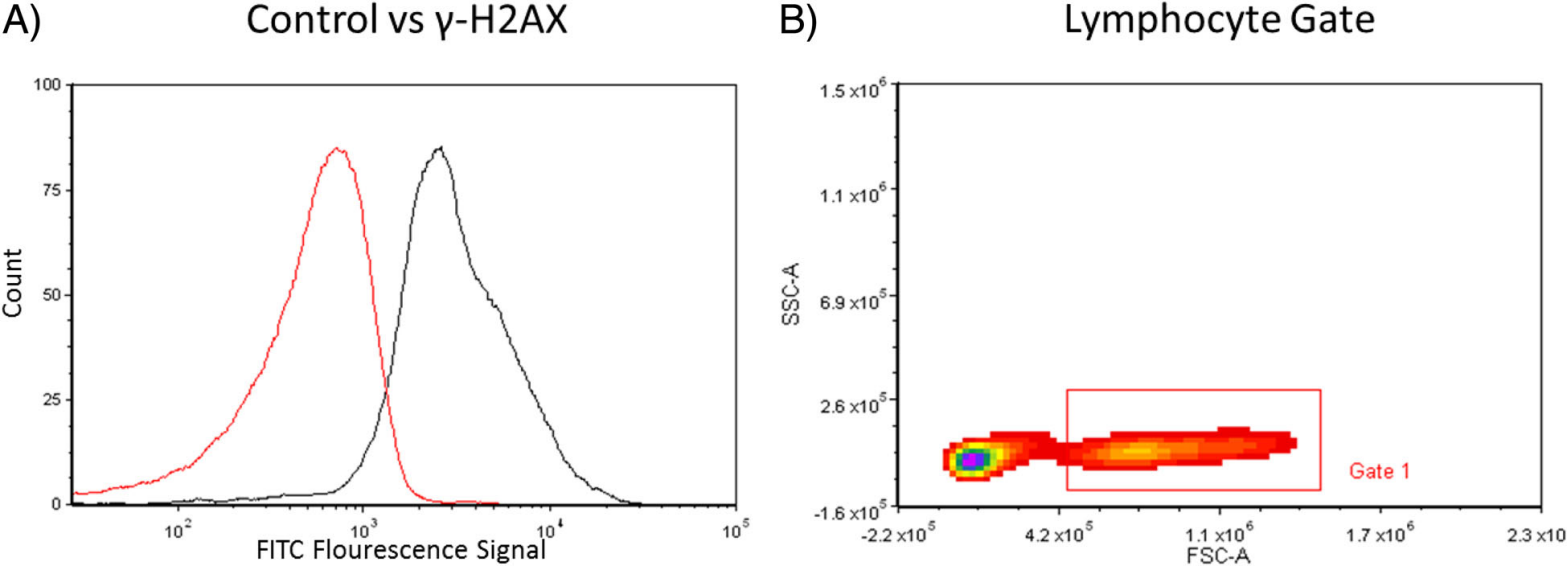
Representative gating for γ-H2AX assay. **a** Control antibody stained sample vs. γ-H2AX stained sample (gated on P1); **b** Lymphocyte Gate (P1)

**Fig. 2:**
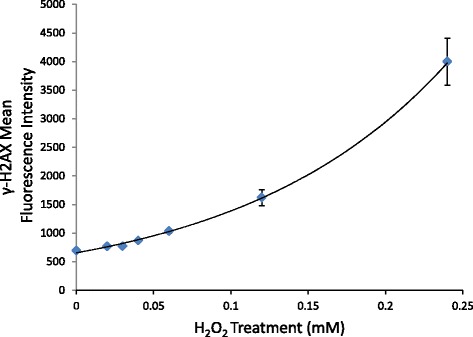
Mean fluorescence intensity of γH2AX values (and standard error bars) in lymphocytes treated with hydrogen peroxide. Cells were isolated from one volunteer on 3 consecutive days, treated and stained for γ-H2AX

Methodological details on processing of peripheral blood samples are described in Additional file 2. Lymphocyte samples (*n* = 80) were tested to evaluate levels of γH2AX. This method was optimized by treating fresh lymphocytes, in triplicate from one volunteer who was not a roofer, with various amounts of H_2_O_2_ (0.02-0.24 mM) and freezing via the same method described above. Frozen samples were thawed in a 37 °C water bath (VWR, Radnor, Pennsylvania) and 500,000 cells were added to wells in a round-bottom 96 well plate (Nunc, Roskilde, Denmark). Freezing media was removed and cells were washed 3 times with PBS. All washes and buffer removals involved a 600 g spin for five minutes at room temperature. Cells were then fixed with 200 μL BD Cytofix fixation buffer (BD Biosciences) and incubated for 15 min at room temperature. Next, the fixative was removed, and cells were washed twice with 200 μL of PBS. Cells were then permeabilized with 200 μl −20 °C Perm Buffer III (BD Biosciences) for 5 min at RT. After one wash in 200 μL of 1x perm/wash buffer (BD Biosciences), 200 μl of 1x stain buffer (BD Biosciences) was added to each well to block non-specific binding. After 20 min at room temperature the cells were washed two times with 200 μl of Perm/Wash buffer. Next, 100 μl stain buffer and 5 μl BD antibody (557782, Alexa Fluor 488 Mouse IgG1 k Isotype control and 560445, Alexa Fluor 488 Mouse anti- γH2AX IgG1 κ) were added and cells were incubated for 60 min at room temperature in the dark. After antibody removal, wells were washed three times with 200 μl of 1x perm/wash buffer. Finally, the cells were resuspended in 300 μl FACS fix (1x PBS with 0.1 % sodium azide and 4 % formaldehyde) and read with CFlow Plus software on a C6 flow cytometer (Accuri Cytometers, Ann Arbor, Michigan). All samples were run in triplicate and results are given in mean fluorescence intensity (MFI) of the lymphocyte gated FL1 channel (Fig. 1). Control antibody values were subtracted from γH2AX antibody values to determine final MFI for each sample. Positive control samples were obtained by treating volunteer lymphocytes with hydrogen peroxide (H_2_O_2_). Plots for publication were made using the FCS Express4 Flow Research Edition software. Figure 2 presents γH2AX MFI in peripheral blood lymphocytes of one laboratory volunteer (not a roofer and nonsmoker) collected over three consecutive days and treated with varying doses (0.02-0.25 mM) of H2O2 on each day. We observed a positive dose–response between H2O2 treatment and γH2AX MFI that ranged from 692 MFI at zero H2O2 to 4000 MFI at 0.25 mM H2O2 (Fig. 2).

### Statistical analyses

All statistical analyses were conducted using SAS system software (version 9.4; SAS Institute, Cary, NC) at a significance level of 0.05. All tests were performed after (natural) logarithmic transformation of urinary analytes (PAH metabolites, creatinine, 8-OHdG), γH2AX and PAH levels (air and dermal wipe) to satisfy the normality assumption, and data were summarized as geometric means (GMs) and geometric standard deviations (GSDs). Average levels of naphthalene and pyrene in personal breathing zone samples or on dermal wipes were compared between the two study days or between smokers and nonsmokers for each day, using Student’s t-tests. Specific contrasts (with alpha = 0.05) were applied to test for differences in log-transformed mean levels of urinary biomarkers and DNA damage measures by sampling period (before and after the work-shift on first and second day) and cigarette smoking status. For this purpose, a two-way analysis of variance (ANOVA) procedure was applied for each day separately. For each sampling day, Pearson’s correlation coefficients (with 95 % CI) were used to measure the strength of association between different pairs of exposure and biomarker measurements.

In personal air samples using filters (FLT) 42.5 % of naphthalene measurements and 35 % of benzo(e)pyrene measurements were below limit of detection (LOD). For personal air XAD samples 42.5 % of pyrene was below LOD, while naphthalene was detected in all of the XAD samples. For dermal wipes, 3.5 % for naphthalene samplers were below LOD and pyrene was detected in all of the samples. Measurements of γH2AX (lymphocytes) and 8-OHdG (urine) were above detection limit for all of the samples. For urinary biomarkers: 1-OHPyr was below LOD in 24 % of the samples while 1-OHNap was below LOD in one urine sample (1.2 %). 2-OHNap and creatinine were both detected in all of the urine samples. For samples that were below the LOD, a proxy measurement was assigned using the value of LOD/√2 before statistical analyses [57].

Information for a number of general and work-related variables was collected via questionnaires (Additional file 1). The general variables included the following: cigarette smoking status, demographic variables (age, race/ethnicity), possible dietary exposure to PAHs (number of servings for consumption of grilled, broiled or smoked meat/fish/chicken within the last 24 h), and number of alcoholic drinks consumed within the last 24 h. The work related variables included the following: removal of old roof (yes/no), application of new roofing (yes/no), work with hot asphalt (yes/no), work as kettleman (yes/no), exposure to diesel exhaust during day’s work (yes/no), percent of time when gloves or a face mask was used, if clothing was short-sleeved or long-sleeved, if hands were washed anytime during work prior to sampling (yes/no), if any solvents or other chemicals were used to clean skin (yes/no), and if the roofer experienced skin burn due to contact with hot asphalt (yes/no).

Repeated-measures general linear mixed modeling (PROC MIXED) was used to examine associations between DNA damage measures (γH2AX and 8-OHdG) and sampling period (before/after work on two separate sampling days) adjusting for confounders. For the general linear mixed models only confounders with values at the four different sampling periods were considered. Candidate variables that were considered amounted to eight variables for the model of γH2AX (urinary biomarkers: 1-OHNap, 2-OHNap, and 1-OHPyr; dermal levels of naphthalene and pyrene; sampling period, age, and cigarette smoking status), and nine variables for the model of urinary 8-OHdG (urinary biomarkers: 1-OHNap, 2-OHNap, and 1-OHPyr; dermal levels of naphthalene and pyrene; sampling period, age, and cigarette smoking status, and urinary creatinine), plus the two-way interactions between urinary biomarkers and cigarette smoking in both models. The most likely candidate variables were screened as follows. First, DNA damage measures were regressed on each covariate separately and variables that suggested significant contributions (*p* < 0.10) were retained. Backward selection of all retained independent variables and their plausible two-way interactions were used to achieve final models (using a significance level of *p* < 0.05). Multivariable models had the general form:

$$ {Y}_{ij}=\alpha +{b}_i+{\displaystyle \sum_{k=1}^p}{\beta}_k{X}_{ik}+f\left( perio{d}_j\right)+{\varepsilon}_{ij} $$

where *Y*_*i*_ represents the subject-specific mean of log-transformed levels of γH2AX (or 8-OHdG) for the *i* ^*th*^ subject at sampling period *j* ^*h*^, *α* is the intercept representing the average level of *Y*_*i*_ when all independent variables are zero for an average worker, b_*i*_ is the random intercept for subject i that captures the heterogeneity between individuals, *β*_*k*_ is the regression coefficient for the *k* ^*th*^ independent variable *X*_*ik*_ for the *i* ^*th*^ subject, and *ε*_*ij*_ is the error term. Given the sample size of 80 measurements from 20 individuals, a linear mixed model with about 4–6 predictors will likely be a stable model. Estimates of the percentage of variance explained by each of the significant covariates in the linear mixed models were calculated using the conditional and marginal formulas of R^2^ [58]. The intraclass correlation coefficients (ICC) were estimated for final models of each DNA damage measure, using ICC = between-subject variance/(between-subject variance + within-subject variance).

## Results

Levels of DNA damage markers were at higher concentrations in samples collected after work when compared to those observed before work. The overall difference between post-shift and pre-shift levels was 1.7-fold for urinary 8-OHdG (3972.3 and 2367.3 μg/g creatinine, respectively, *p*-value < 0.0001 for difference). When divided by smoking status, post-shift levels of 8-OHdG remained to be higher than those observed pre-shift in nonsmokers (3714.5 and 2368.5 μg/g creatinine, respectively) and in smokers (4146.4 and 2344.9 μg/g creatinine, respectively), difference was statistically significant for both groups (*p* < 0.05). The overall difference between post-shift and pre-shift levels was smaller for γH2AX (1157.7 and 1229.7 MFI, respectively, *p* = 0.048 for test of difference) and remained small in nonsmokers (1164.4 and 1107.7 MFI, respectively, *p* = 0.2) and in smokers (1286.9 and 1188.0 MFI, respectively, *p* = 0.09).

**Fig. 3:**
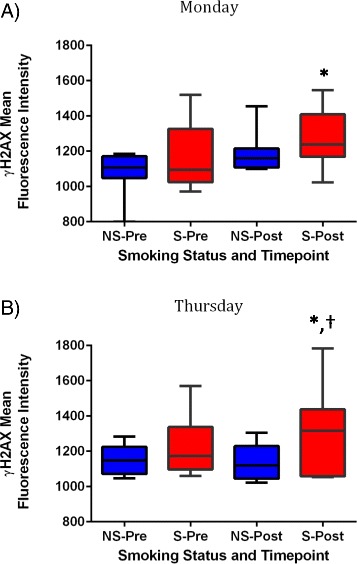
Mean fluorescence intensity values of γ-H2AX for nonsmokers (*n* = 8) and smokers (*n* = 12) by study day and sampling time (pre and post work-shift). **a** Monday **b** Thursday. NS = nonsmokers, S = smokers, Pre: pre-shift, Post: post-shift. * *p* < 0.05 when compared to means of nonsmokers, pre-shift within same sampling day. †*p* < 0.05 when compared to levels of non-smokers after work within same study day

Figure 3 presents average γH2AX MFI in peripheral blood lymphocytes collected from roofers during the study. Average pre-shift MFI was 1081 on Monday and 1150 on Thursday among nonsmokers. On Monday, we observed an increase in γH2AX MFI over the work shift in both smokers and nonsmokers (Fig. 3a). On Thursday, however, only levels in smokers increased during work hours while levels of γH2AX in nonsmokers remained similar after work (Fig. 3b). When average levels were compared using two-way ANOVA, the highest levels of γH2AX MFI were observed in smokers after work, while the lowest levels were observed among nonsmokers before work (Fig. 3).

Among PAHs in the gaseous phase (obtained via XAD adsorbent tubes), naphthalene was the most abundant PAH, quantified in all of the samples. Pyrene on the other hand, was only quantified in 57.5 % of the XAD samples. For PAH measurements obtained from filters (representing particulate phase) the most abundant compound was benzo(e)pyrene quantified in 65 % of all samples, followed by naphthalene quantified in 57.5 %. Only 35 % of filter samples had quantifiable levels of pyrene. Table 1 presents geometric mean levels of selected PAHs by study day. Cigarette smokers had higher exposure levels for most PAHs, particularly on Thursday, but the differences between levels in smokers and nonsmokers were not statistically significant (see Additional file 3: Table S1).

**Table 1:** Geometric means (and geometric standard deviations) of PAH measures in personal air samples (ng/m^3^) collected over the work hours (A) and PAH levels in dermal wipes (μg/wipe) collected before and after work (B)

A. Airborne exposure measurements (*n* = 40)		
Naphthalene (ng/m^3^, XAD)	361.4 (2.9)	
Pyrene (ng/m^3^, XAD)	2.3 (7.5)	
Naphthalene (ng/m^3^, FLT)	0.78 (2.7)	
Benzo(e)pyrene (ng/m^3^, FLT)	3.5 (8.9)	
B. Dermal exposure measurements collected before (*n* = 40) and after the work shift (*n* = 39)		
PAH (μg/wipe)	Before	After
Naphthalene	0.32 (1.3)	0.32 (1.3)
Pyrene	0.15 (1.5)	0.35 (2.4)^*^

*FLT* PAHs in particulate phase from filters, *XAD* PAHs in gaseous phase from XAD adsorbent tubes

^*^ *p* < 0.0001 when compared to before work levels

Levels of naphthalene on dermal wipes did not differ significantly before or after the work on both study days (Table 1B). Levels of pyrene, however, significantly increased over work hours (Table 1B).

Table 2 presents geometric mean levels of urinary biomarkers before and after work by week day. On Monday, levels of 1-OHPyr and 8-OHdG were higher in the afternoon (3- and 2-fold, respectively), when compared to levels observed in morning samples. Urinary 1-OHPyr levels increased during work on Thursday as well, but this was not observed for urinary 1- and 2-OHNap (Table 2).

**Table 2:** Geometric means (and geometric standard deviations) of urinary biomarkers (μg/g creatinine) and of γH2AX (from lymphocytes) in samples collected before and after work on both study days

Biomarker	Monday		Thursday	
	Before (*n* = 20)	After (*n* = 20)	Before (*n* = 20)	After (*n* = 19)
1-OHNap	13,766.6 (4.3)	24,343.0 (3.2)	17,500.8 (2.8)	18,769.7 (4.5)
2-OHNap	54,176.4 (2.0)	73,130.4 (2.5)	73,130.4 (2.0)	73,130.4 (2.1)
1-OHPyr	706.3 (3.7)	2100.6* (3.0)	1032.8 (4.5)	1790.0 (3.7)
8-OHdG	2186.4 (1.8)	4105.2* (1.4)	2565.7 (1.4)	3866.1* (1.5)
γH2AX (MFI)	1118.8 (1.2)	1224.1* (1.1)	1188.0 (1.1)	1236.4 (1.2)

*p < 0.05 when compared to pre-shift levels within the same day. *MFI* mean fluorescence intensity

All urinary biomarkers were higher after work on Monday in both smokers and nonsmokers, but the post-shift to pre-shift difference was statistically significant only for urinary 1-OHPyr and 8-OHdG in smokers (Additional file 3: Table S2). On Thursday, levels of urinary biomarkers increased over work hours in nonsmokers, with a statistically significant difference observed for urinary 8-OHdG (*p* = 0.005, Additional file 3: Table S2). For smokers, the pattern was different; highest levels of urinary 1- and 2-OHNap were observed in the morning and diminished during work hours (Additional file 3: Table S2).

Pearson correlation coefficients between pairs of log-transformed values of PAHs observed in air, dermal wipes, and in urine and post-shift measures of DNA damage are presented in Additional file 3: Table S3. While measurements of γH2AX correlated with those of urinary 1- and 2-OHNap on Thursday, no other significant correlation was observed between measures of exposure, metabolites or DNA damage (Additional file 3: Table S3).

Table 3 presents results of final mixed-effects linear regression models of γH2AX and urinary 8-OHdG in samples collected from 20 roofers at four different sampling periods (period 1 = Monday before work, 2 = Monday after work, 3 = Thursday before work, 4 = Thursday after work). None of the dermal exposure measures and urine biomarkers were significant predictors of either γH2AX or of 8-OHdG in the mixed-effects models and were not retained in final models. For measures of γH2AX, sampling period and cigarette smoking status were significant predictors. Being a smoker corresponded to an 8.5 % increase in logged γH2AX measurements (Table 3). The percentage of the between-worker variation of γH2AX in the log scale was 35.3 % of the total variance (intraclass correlation coefficient = 35.3 %, Table 3).

**Table 3:** Linear mixed effects models of DNA damage measures in roofers (samples from 20 workers at 4 sampling periods, *n* = 79)

	Estimate (SE)	*p*-value
A) Model for γH2ax^a^ (lymphocytes)		
Fixed effects		
Intercept	6.97 (0.04)	<0.0001
Smoker (yes/no)	0.085 (0.04)	0.04
Period 2 (Monday, after work)	0.09 (0.03)	0.008
Period 3 (Thursday, before work)	0.06 (0.03)	0.06
Period 4 (Thursday, after work)	0.1 (0.03)	0.006
Period 1 (Monday, before work)	0 (ref.)	
Random effects		
Between-subject variance	0.006 (0.002)	0.02
Within-subject variance	0.011 (0.002)	<0.0001
Intraclass correlation coefficient %	35.3	
B) Model for 8-OHdG^a^ (urine)		
Fixed effects		
Intercept	6.99 (0.19)	<0.0001
Urine creatinine^a^ (g/L)	0.68 (0.08)	<0.0001
Period 2 (Monday, after work)	0.55 (0.12)	<0.0001
Period 3 (Thursday, before work)	0.13 (0.12)	0.27
Period 4 (Thursday, after work)	0.50 (0.12)	0.0002
Period 1 (Monday, before work)	0 (ref.)	
Random effects		
Between-subject variance	0.007 (0.02)	0.32
Within-subject variance	0.14 (0.03)	<0.0001
Intraclass correlation coefficient %	4.8	

^a^ Analyses are done after (natural) logarithmic transformation

Based on our model (see Table 3) the predicted mean value of γH2AX in samples collected on a Monday afternoon from roofers who are smokers can be calculated as follows: ln(γH2AX) = 6.97 (intercept) + 0.085 (smoker) + 0.09 (Period 2) = 7.14 and thus γH2AX =1261.4 MFI.

For γH2AX, according to the conditional R^2^, 44.3 % of the variation was explained by sampling period alone whereas 45.4 % was explained by period and smoking status; the marginal R^2^ estimates for these models were 7.6 and 16.4 %, respectively (data not shown).

In models of urinary 8-OHdG measurements, cigarette smoking was not a significant predictor, but urine creatinine had a great impact on the levels and was kept in final models. Sampling period also remained a significant predictor. When compared to Monday morning, levels collected on Monday afternoon corresponded to 55 % higher levels of urinary 8-OHdG, and levels collected at the end of the week (period 4) were increased by 50 %. The percentage of the between-workers variation of 8-OHdG in the log scale was 4.8 % of the total variance (Table 3).

Based on our model (see Table 3) the predicted mean value of 8-OHdG in urine samples collected on Monday afternoon from roofers with an average urine creatinine value of 0.1 g/L can be calculated as follows: ln(8-OHdG μg/L) = 6.99 (intercept) + 0.68 * 0.1 (creatinine, g/L) + 0.55 (period 2) = 7.608 and thus, urine 8-OHdG = 2014.3 μg/g creatinine.

For the analysis of logged 8-OHdG, according to the conditional R^2^, 18.8 % of the model variation was explained by period alone whereas 56.9 % was explained by period and urine creatinine; the marginal R^2^ estimates for these models were 8.2 and 54.7 %, respectively (data not shown).

## Discussion

We have recently shown that urinary 8-OHdG is a promising biomarker reflecting early effects of occupational exposures to PAHs during a single work day [38]. Here we expand our work to include another distinct measure of DNA damage, γH2AX. While the assessment of γH2AX has been previously used in experimental studies with human cell lines [59–66], this is the first attempt to link human occupational exposures to increased levels of γH2AX. This study is also the first to apply high-throughput flow cytometry to quantify γH2AX in peripheral lymphocytes of workers making it a more feasible option for population-based research. Our results support the idea that work with hot asphalt contributes to higher levels of oxidative DNA damage and DNA double-strand breaks. This effect was more obvious among nonsmokers and on the first week day.

In this study, sampling period, reflecting four different time points within one workweek, was an important predictor of both DNA damage markers. Other important predictors were cigarette smoking for γH2AX and urinary creatinine for urinary 8-OHdG. Our results once again support that urinary 8-OHdG is highly affected by urine dilution. This is an important concern since many times roofers are exposed to heat and can be dehydrated during the course of a single work day.

We also observed that about 35.3 % of the unexplained variance of γH2AX was between subjects, while this number was only 4.8 % for urinary 8-OHdG. The proportion of within-subject variance appears larger for urinary 8-OHdG. These two measures cannot be directly compared as they reflect different types of DNA damage (while γH2AX is a measure of DNA double-strand breaks, 8-OHdG is a measure of oxidative DNA damage) and are measured in different biological media (γH2AX from lymphocytes and 8-OHdG in urine). However, a low ICC value reflects high within-individual variation of a biomarker and is a sign of poor reproducibility [67]. Other studies also reported high intra-individual variation for urinary 8-OHdG, including in urine samples collected over 24 h [68–70]. Urinary 8-OHdG is influenced by many individual factors, such as cigarette smoking, dietary factors, or diurnal fluctuations, which may explain some if this high intra-individual variation. Cigarette smoking was not a significant predictor of 8-OHdG levels in our study, but it is possible that other individual factors may have contributed to its variation.

Levels of pyrene on dermal wipes were higher after work when compared to before work measures. This increase was not observed for naphthalene. Consistently, urinary 1-OHPyr levels significantly increased over work hours on Monday, while the increase in urinary naphthalene metabolites was small. Naphthalene is the most abundant PAH in many environments and naphthalene based biomarkers can potentially increase sensitivity of assays. However, our results in this population suggest that environmental influences and cigarette smoking can overwhelm those of occupational exposures to naphthalene. Results of this study are consistent with our previous findings that urinary 1-OHPyr is a promising biomarker of occupational exposures in roofers and that dermal contact can be an important exposure route [38].

Urinary metabolites of PAHs, particularly urinary 1-OHPyr, are established biomarkers of occupational exposures. Overall, levels of PAH exposures in this study were lower than previous reports in asphalt exposed workers [71, 72]. While naphthalene metabolites were comparable to levels observed in our prior work with roofers, post-shift levels of urinary 1-OHPyr were much lower in the current study [38]. Here, the highest concentration of urinary 1-OHPyr was observed among nonsmokers in post-shift samples collected on Monday (333.6 ng/l). This is comparable to pre-shift levels of nonsmokers (213 ng/l) observed in our prior work [38] where the highest average levels of post-shift 1-OHPyr was measured as 1002 ng/l in smokers [38]. In fact, levels of urinary 1-OHPyr in this study are comparable to those observed in the general population [73, 74].

Despite the increase in urinary biomarkers during work hours, we did not observe consistent correlations between measures of exposure, urinary metabolites, and DNA damage, making it difficult to reach a final conclusion on the association between exposure and biomarkers. However, this is possibly related to the overall low occupational exposures observed during the study period. Consistently, Monday morning levels of urinary 8-OHdG were are approximately 36-fold lower than those observed in our previous study in roofers [38].

Cigarette smoking is an important factor to consider when analyzing γH2AX. The correlation between γH2AX and urinary metabolites of naphthalene on the second study day was possibly due to their common association with cigarette smoking. This is supported by the fact that levels of urinary 1- and 2-OHNap among smokers were much higher before the work shift on Thursday when compared to levels after the work. It is tempting to limit future evaluations to nonsmokers. However, considering the high proportion of smokers [11] and the challenges of recruiting participants, such a restriction would be impractical in the roofer population. We believe that the best approach is to observe and record cigarette smoking habits and evaluate it as a possible confounder in final analyses.

There are some limitations of this study that constrain the interpretation of results. The most important limitations are the low levels of exposure observed during the study period and the small number of participants. Repeated sampling at four different time points, however, provided us with a larger effective sample size. We also used ANOVA to test for differences between measurements conducted before and after the work shift without adjustment for repeated measures. While before and after-work samples do not reflect identical conditions, they are also not independent. However, this was only a preliminary approach that has been addressed by the use of linear mixed models where the specific contrasts of interest have been tested. We also recognize that we are conducting some hypothesis tests without adjustment for multiple comparisons, which in turn might produce a few false positive associations.

Another limitation is the widespread environmental exposure to PAHs and other toxicants that cause DNA damage. 1-OHPyr has been widely viewed as the gold standard biomarker of PAH exposures [75–77]. Naphthalene is present mostly in the gaseous phase and is mainly absorbed through inhalation; pyrene, on the other hand exists both in gas and particulate phase and can be absorbed through inhalation and dermal contact [21]. Usefulness of naphthalene metabolites can be limited if non occupational exposures are common or if occupational exposure is predominantly through the dermal route, in which case urinary 1-OHPyr may serve as a better biomarker.

The PAH biomarkers studied here reflect short-term exposures. The estimated elimination half-lives for urinary 1- & 2-OHNap and 1-OHPyr are around 4 h [78] and 13 h [79], respectively. Because the focus of this study is to link short-term PAH exposures to short-term markers of DNA damage, the rapid elimination of these biomarkers is not expected to restrict our study results.

While γH2AX is an early response to genotoxic insults, a number of non-occupational factors can contribute to the DNA double strand breaks (DSBs), such as ultraviolet light (UV), environmental chemicals, and even endogenous triggers of DNA damage. Tobacco smoke, a common source of PAHs, is also a potent inducer of DSBs. With the high number of factors influencing γH2AX response, it is important to distinguish between baseline levels in the general population and γH2AX kinetics following specific exposures. Two types of γH2AX foci have been reported previously: the fast transient γH2AX foci associated with rapid repair which takes place within minutes or hours, and the residual foci that persist for several days or months [80]. The majority of DSBs are repaired during the fast phase usually within minutes, and only about 20 % are repaired during the slower phase [80]. The persistent foci may be the result of slow repair or they may reflect unrepaired damage. Individual factors, such as gender, hormonal response, ethnicity and race, and life style factors (smoking and alcohol consumption), age, and age related diseases such as hypertension or cataracts [81] may further influence γH2AX repair kinetics [82]. It is also possible that long term occupational exposures induce endogenous DSB formation and contribute to the persistent γH2AX response. When designing our study we aimed to evaluate exposure and DNA damage at four different time points. This was based on prior knowledge that pre-shift biomarker levels will be higher towards the end of the workweek than those measured at the beginning [83]. An additional benefit of keeping the four separate time points was the observation that behavioral or individual factors, such as cigarette smoking, can further influence DNA damage levels during the workweek.

## Conclusion

Our overall goal was to explore the usefulness of γH2AX as a possible marker of DNA damage in workers exposed to PAHs using a high throughput flow cytometry assay. One of our evaluation criteria was its association with exposure data, which we could not observe for γH2AX. Urinary 8-OHdG, a commonly used marker of DNA damage, was also not associated with exposure levels in this group. It is possible that the relatively low levels of exposures may have impacted our analyses. As a second criterion, we evaluated the between- and within-subject variation of γH2AX. Here we observed that γH2AX has a smaller within-worker variation when compared to urinary 8-OHdG. Our analyses also confirmed that baseline values of γH2AX are easily detectable in this population, using an inexpensive method such as flow cytometry. Despite the lack of association with exposure data, we propose that γH2AX is a sensitive biomarker of early DNA damage related to occupational exposures. The low within-subject variation, easy and high throughput methodology makes γH2AX a feasible alternative in epidemiology studies. We perceive the need for additional studies to understand baseline values of γH2AX, between- and within-individual variation in different study populations, and the impact of developmental and degenerative diseases, as well as dietary, environmental, and life-style factors on this promising biomarker [80]. This will also help develop criteria to distinguish between transient and persistent γH2AX foci.

## Additional files

Additional file 1: Before and after work questionnaires. (PDF 238 kb)
Additional file 2: Supplementary Methods. (DOCX 22 kb)
Additional file 3: Table S1. Geometric means (and geometric standard deviations) of PAH measures in personal air samples (ng/m^3^) by study day and smoking status. **Table S2.** Geometric means (and geometric standard deviations) of urinary biomarkers (μg/g creatinine) in samples collected before and after work on both study days by smoking status. **Table S3.** Correlation between PAH exposure and biomarker data. Pearson correlation coefficients (and *p*-values) of log transformed measurements are presented for Monday (upper clear cells) and Thursday (lower grey shaded cells) post-shift samples. Urinary PAH metabolites and 8-OHdG are adjusted for urine creatinine. FLT = filter/particulate phase, XAD = adsorbent tube/gaseous phase, DERM = dermal wipe samples. (DOCX 36 kb)

## Abbreviations

1-OHNap: 1-hydroxynaphthalene
1-OHPyr: 1-hydroxypyrene
2-OHNap: 2-hydroxynaphthalene
8-OHdG: 8-hydroxy-2′-deoxyguanosine
ANOVA: Analysis of variance
BaP: benzo(a)pyrene
ELISA: Enzyme-linked immunosorbent assay
FLT: Filter
GC/TOFMS: Gas chromatography with time of flight mass spectrometry
GC-MS: Gas chromatography/mass spectrometry
GMs: Geometric means
GSDs: Geometric standard deviations
LOD: Limit of detection
MFI: Mean fluorescence intensity
NIOSH: National Institute of Occupational Safety and Health
OH-PAH: Hydroxylated PAH
PAHs: Polycyclic aromatic hydrocarbons
PBMCs: Peripheral blood mononuclear cells
PBS: Phosphate buffered saline
ROS: Reactive oxygen species
SIM: Selected ion monitoring
SPE: Solid phase extraction
γH2AX: phosphorylated histone H2AX
