# Supplementary material for: Short-term markers of DNA damage among roofers who work with hot asphalt
Source: Environ Health. 2016 Oct 20;15:99. doi: 10.1186/s12940-016-0182-4 (PMC5072307; doi:10.1186/s12940-016-0182-4)
Supplement: Additional file 1: — Before and after work questionnaires. (PDF 238 kb) [file 12940_2016_182_MOESM1_ESM.pdf]

Colorado School of Public Health

Department of Environmental and Occupational Health

COMIRB # 12-0443
Study Title: Polycyclic aromatic hydrocarbons exposure and DNA damage in roofers
PI Name: Berrin Serdar

CONTRUCTION WORKERS EXPOSURE TO ASPHALT STUDY

APPROVED
MAY 14 2013
COMIRB

BEFORE-WORK QUESTIONNAIRE

Location of study site:____________ Date:______________

Participant study identification number:___________________

---

1. What is your current height and weight?

Height (ft.-in.):______________ Weight (lbs):____________

2. What is your age and gender? Age (years):_______

Gender: ____Male ____Female

3. What is your ethnic background?

____Black or African American
____White
____Hispanic or Latino
____American Indian or Alaska Native
____Pacific Islander or Hawaiian
____Asian
____Other. Please describe:___________________________________

4. Do you have any medical problems that you are aware of? ___Yes ___No

If yes, please describe your medical problem(s):

____

____

5. Are you currently taking any medication(s): _____Yes ___No

If yes, please give the names of the medication(s): ____________________________________

____

6. Do you smoke cigarettes?

___Yes ___No

7. Do you use any other tobacco products (cigars, pipes, chewing tobacco, etc.)?

___Yes ___No

If yes, please specify: ______________________________

8. How many cigarettes or any of the following tobacco products do you smoke on average (daily or monthly)? Please select those that apply.

_____cigarettes/day _____cigars/day _____pipes/day

_____cigarettes/month _____cigars/month _____pipes/month

If the participant lists any tobacco products other than cigarettes, cigars or pipes (e.g., chewing tobacco) please specify the daily/monthly consumed amount for those products:

____

9. How many cigarettes (or other tobacco products) did you smoke within the last 24 hours?

_______cigarettes
_______cigars
_______pipes
_________other tobacco products

10. When was the last time you smoke any cigarettes or other tobacco products (such as cigars, pipes, etc.)?

Time: ____________ Date: ____________

11. If you do not smoke currently have you ever smoked in the past?

__Yes __No

12. If you smoked in the past, when did you quit smoking? ____________________

13. Are you exposed to cigarette smoke at home (are others in your household smoking at home)?

__Yes __No

14. During the last 24 hours, have you been in any environments where there was cigarette smoke?

__Yes __No

15. How frequently do you consume alcoholic beverages?

__Never
__Less than once a month
__Once a month
__2-3 days/month
__1-2days/week
__3-4 days/week
__Every day

16. If you consume alcoholic beverages, please select the drinks that you consume most frequently

__Beer
__Wine
__Liquor (e.g., brandy, gin, vodka, tequila, whisky, rum)

17. On those days when you consume alcoholic beverages, how many drinks do you usually consume in one sitting? Please select those that apply.

__________drinks

18. Have you consumed any alcoholic beverages within the last 24 hours (since yesterday morning)?

__Yes __No

If yes, please describe the type and amount of the alcoholic drink consumed:

____

19. Have you consumed any of the following food items during the last 2 days? If yes, please write how many servings (4-6 ounce) you consumed for each day.

	Number of servings (4-6 ounce) consumed		
	This morning	Yesterday	The day before yesterday
Grilled or barbequed meat			
Grilled or barbequed chicken			
Grilled or barbequed fish			
Fried chicken			
Fried fish			
Fried meat			
Smoked meat			
Smoked chicken			
Smoked fish			
Other smoked products (e.g., smoked deli meat)			

20. Do you have any of the following skin problems?

__Skin irritation

__Rash

__Burn

__Dermatitis

__History of skin allergies

21. Did you apply any skin lotion, moisturizer, or medicine on your hands today?

__Yes __No

If yes, please describe the product you applied:

____

22. Did you perform any construction/roofing work during the last 48 hours (yesterday or the day before yesterday)?

__Yes __No

If yes, please describe the work and date you worked:

____

23. During the last 48 hours (yesterday and the day before), have you come into contact with any of the following materials or chemicals? If the answer is yes, please describe the contact.

Asphalt __Yes __No

____

Coal tar (can be used in roofing, driveway sealers, dandruff shampoo, etc.)
__Yes __No

____

Diesel or gasoline fuel __Yes __No

____

Exhaust (from diesel or gasoline) __Yes __No

____

Other chemicals such as solvents, oils, paints, coating, etc.

__Yes __No

If yes for other chemicals, please describe the source:

____

____

____

____

THANK YOU!

Department of Environmental and
Occupational Health

COMIRB # 12-0443
Study Title: Polycyclic aromatic hydrocarbons exposure and DNA damage in roofers
PI Name: Berrin Serdar

APPROVED
MAY 14 2013
COMIRB

CONTRUCTION WORKERS EXPOSURE TO ASPHALT STUDY

AFTER-WORK QUESTIONNAIRE

Location of study site:________________________ Date:______________

Participant study identification number:____________

Information to be obtained from the contractor:

Type of asphalt used in this roofing project:

____

If old roof was removed, did the torn-off roof contain coal tar? ___Yes ___No

1. What is your current job title? _________________________________

2. How long have you been in this occupation? ____________________________

3. Did you smoke any cigarettes (or other tobacco products) since your first interview this morning?

___Yes ___No

If yes, please tell us how many cigarettes (or other products) did you smoke since this morning?

______cigarettes
______cigars
______pipes
______other tobacco products.

If other tobacco products, explain what type:___________________________________

4. How long ago did you smoke your last cigarette (or other tobacco products)?

_____minutes ago _____hour(s) ago

5. Did you smoke while wearing the air sampler?

___Yes ___No

6. During your work today have you been near someone else who smoked?

___Yes ___No

If yes, please tell us if this occurred while wearing the air sampler:

___Yes ___No

7. Since this morning did you eat any of the following food items. If yes, please write how many servings (4-6 ounce) you ate.

	Number of servings since this morning
Grilled or barbequed meat	
Grilled or barbequed chicken	
Grilled or barbequed fish	
Fried chicken	
Fried fish	
Fried meat	
Smoked meat	
Smoked chicken	
Smoked fish	
Other smoked products (e.g., smoked deli meat)	

8. What tasks did you perform today? Please check all that apply.

___Tearing off old roofing material

___Application of new roofing material

___Worked with hot asphalt

___Worked as kettleman (worked with kettle to melt asphalt)

___Other tasks

Please list any other tasks that you performed:

____

____

____

____

____

9. What type of materials were you exposed to during today's work? Please check all that apply and describe the source and how you were exposed.

Asphalt ___Yes ___No

____

Coal tar ___Yes ___No

____

Asbestos ___Yes ___No

____

Exhaust (diesel/gasoline) ___Yes ___No

____

Gasoline or fuel ___Yes ___No

____

Silica __Yes __No

Formaldehyde __Yes __No

Solvents __Yes __No

Paints __Yes __No

Adhesives __Yes __No

Caulks/Sealants __Yes __No

Please list any other chemicals that you were exposed to during your work today.

---

10. What type of roofing did you work with today:

__Built up roofing
__Asphalt shingles
__Roll roofing
__Modified bitumen systems
__Other

If other, please describe:

---

11. Which of the following protective equipment(s) did you use during today's work? For those that were used, please indicate what percentage of time you used them during work.

Protective equipment	Time used (% of work)
___Gloves	________________
___Face mask	________________
___Other (please describe):____________	________________

____

12. If you used gloves during work, please describe what type of gloves these were:

____

13. If you used gloves, were they heat resistant?

___Yes ___No ___Don't know

14. If you used a face mask or respirator, please describe the type you used (e.g., NIOSH approved dust mask, ½ face with cartridges, etc.):

____

____

15. What type of clothing did you wear during today's work? Please check all that apply.

___Regular daily clothing
___Regular clothing, but used during this work
___Overalls
___Protective shoes
___Others

Please describe if other protective clothing used:

____

16. Did you wear short or long sleeves during today's work?

___Short sleeves ___Long sleeves ___I had both during work

17. How frequently do you change into clean work clothing or overalls?

__Every day
__Once a week
__Once a month
__Less frequently than once a month

18. How are your overalls cleaned?

__Water laundering
__Dry cleaning
__Other. Please describe if other: ______________________________

19. Do you usually shower immediately after the work-shift?

___Yes ___No

20. Did anything extraordinary happen during today's work that might increase your exposure to chemicals (e.g. accident, spill, etc.)

___Yes ___No

If yes, please describe the event:

____

21. During today's work did your skin come into contact with asphalt?

__Yes __No

If yes, please describe how:

____

____

22. Did you apply any skin lotion, moisturizer, or medicine on your hands since this morning? __Yes __No

If yes, please describe the product you applied:

____

____

23. Did you wash your hands during work today? ___Yes ___No

If yes, please describe how long ago you washed your hands before providing the hand wipes:

____________________ minutes/hours before hand wipe sampling

24. Did you use any solvents or other chemicals to clean your skin today?

___Yes ___No

If yes, please explain what type of cleaner used: ________________________

25. Have you experienced skin burn related to your work with hot asphalt (as a result of contact with hot asphalt)?

___Never ___Once ___More than once

If you experienced skin burn due to contact with hot asphalt, please describe the following:

How long ago did this burn happen?

____

Where was your skin burned?

____

26. Have you experienced skin irritation which you consider to be related to your work?

___Yes ___No

____

THANK YOU!
