# Supplementary material for: Short-term markers of DNA damage among roofers who work with hot asphalt
Source: Environ Health. 2016 Oct 20;15:99. doi: 10.1186/s12940-016-0182-4 (PMC5072307; doi:10.1186/s12940-016-0182-4)
Supplement: Additional file 2: — Supplementary Methods. (DOCX 22 kb) [file 12940_2016_182_MOESM2_ESM.docx]

**Supplementary Methods**

**Air PAHs**

Polycyclic aromatic hydrocarbons (PAHs) in ambient air were measured within the breathing zone of workers via personal sampling. Particle-bound PAHs, 4-ring and above, were collected using personal sampling pumps (SKC XR5000) fitted with PM_2.5_ sampling inlets (model 2.05, Mesa Labs, Inc.) and 37 mm Teflon filters. Gas-phase PAHs were collected immediately downstream of the filters using standard adsorbent tubes (XAD-2, 2 section, 75/150 mg sorbent). Sample flow rate was set at 2.7L/min and verified pre- and post-sampling using a primary standard. Subjects were outfitted with special vests to accommodate the sampling train without interference with work activities. After sampling, sorbent tubes were capped, placed in double-sealed storage containers, and refrigerated at -80°C. Filters were pre- and post-weighed according to NIOSH method 0500, transferred to appropriate individual polystyrene cases, and refrigerated at -20°C prior to subsequent PAH analysis. Field blanks were collected each day for both filters and sorbent tubes at a rate of 20% (i.e., one blank for every five samples).

Sorbent tubes were extracted in dichloromethane and analyzed using gas chromatography/mass spectrometry (GC-MS) for 2- and 3-ring PAHs. Data reduction included external calibration for all analytes and representative internal standards of D8-naphthalene, D10-anthracene, D12-chrysene, and D12-benzo(a)pyrene.

PM_2.5_ filters were analyzed via gas-chromatography mass spectrometry for 4-, 5- and 6-ring PAHs such as pyrene, and benzo(e)pyrene. Sample preparation consisted of excision of the filter material from the polymethylpentene support ring, extraction with 4mL dichloromethane, concentration under dry, purified nitrogen to 50μl, and transfer into high recovery autosampler vials. Analysis was done by splitless injection of 2μl onto a 30 m GC column and selected ion monitoring (SIM) for primary and confirmatory ions. Data reduction included external calibration for all analytes and representative internal standards of 4- and 5- ring PAHs. Appropriate quality assurance procedures were employed to establish methodological limits of quantification, to evaluate linearity of the calibration curve, and to examine gas-phase breakthrough of the sorbent tubes.

**PAH Metabolites in Urine**

Automated solid-phase extraction was based on a method developed by Romanoff [[54](#_ENREF_54)]. This method is used for extraction and quantification of nine hydroxylated PAH (OH-PAH) species in urine. It uses enzymatic digestion of urine to create free OH-PAH followed by SPE sample cleanup and GC-MS/MS analysis. Briefly, 3ml urine was enzymatically digested overnight at 37°C with 10μl β-Glucuronidase/Arylsulphatase (Roche Diagnostics, Indianapolis, Indiana) in 5ml of 100mM sodium acetate (NaAc) (pH 5.5). Samples were spiked with internal standard containing 20ng each deuterium labeled OH-PAHs and control spikes at 0.5ng of each OH-PAH (50μl of 0.01ng/μl mixed OH-PAH in methanol or 5μl of 0.1ng/μl mixed OH-PAH standard). Samples were loaded to Bond Elut Focus 60mg/3ml SPE cartridges (Agilent Technologies, Santa Clara, California) and conditioned with 1ml methanol followed by 1ml LCMS grade water. The sample tube was then rinsed with 1ml water, which was then added to the SPE. Following a wash with 3ml of 60% 100mM NaAc in methanol the samples were aspirated for 10 minutes at full vacuum and eluted with 3ml dichloromethane into clean conical culture tubes with 5μl n-dodecane added before elution. The samples were then blown down with nitrogen until only dodecane remained. Finally, 100μl of 1:1 hexane: Bis(trimethylsilyl)trifluoroacetamide (bstfa ) with 0.1% trimethylchlorosilane (tmcs) was added and the samples were incubated at 80°C for 30 minutes, cooled, adjusted to 100μl with hexane and injected (2μl) on the GC-MS/MS.

**Urinary Creatinine**

Frozen urine samples were thawed at room temperature and vortexed prior to analysis. Samples were then diluted 1:10,000 with 0.1% formic acid. A 0.5μl sample was injected into an Agilent 1290 UPLC with a 6460 triple quadrupole mass spectrometer. Creatinine separation was done with an Agilent Eclipse Plus C18 column (2.1x 100mm 3.5um). The mobile phases were 0.1% formic acid (A) and acetonitrile with 0.1% formic acid (B) with a gradient from 10% B to 100% B at 3min. The conditions for the positive ionization source were as follows: nebulizer gas flow of 10L/min at 250°C and 25psi 172 kpa; sheath gas flow of 11/min at 375°C. The ion transitions monitored for creatinine were 114.1.1 → 86/44m/z. Samples were quantitated with linear regression (2-200ng/ml) using Agilent Mass Hunter Quantitation software (v.B.04.01).

**PAH Levels on Dermal Wipes**

Dermal exposure samples were collected using a previously published hand washing method [[55](#_ENREF_55)]. Briefly, 3ml of sunflower oil was applied to one palm and the workers were asked to rub their hands together for 1min. Each worker then wiped the sunflower oil from their hands using creped 8.25” × 8.25” wipes (DuPont^TM^ Sontara^®^) that were kindly provided by Micrex Corporation. Wiping included front and back of the hands and between the fingers. The wipes were then placed in an amber glass vial and stored on ice until transported to the lab. Crepe materials were extracted using 25 ml dichloromethane and analyzed for PAHs using GC/MS. Due to inference from chromatography peaks linked to the sunflower oil, extracts were further analyzed using gas chromatography with time of flight mass spectrometry (GC/TOFMS) for PAH determinations using a previously published method [[55](#_ENREF_55)].

**Blood Processing**

Blood samples were collected in two 8-ml EDTA containing vacutainers (BD Biosciences, Franklin Lakes, New Jersey) at the work site, placed on ice and transported to the University of Colorado Denver Biorepository Center for processing. The blood was then transferred to 15ml tubes and centrifuged at 2000rpm for 20minutes at room temperature. Plasma (5ml) was removed from each tube, aliquoted and stored at -80°C. Tubes with red blood cells (RBCs) and peripheral blood mononuclear cells (PBMCs) were diluted to 10 ml with phosphate buffered saline (PBS), mixed and this mixture was layered on top of 5ml lymphoprep (Stemcell Technologies, Vancouver, Canada) in 15 ml tubes and centrifuged at 2000rpm for 20 min at room temperature. The PBMC layers from matched tubes were put into one 50ml tube, diluted to 50 ml with PBS and centrifuged at 1200rpm for 10 minutes at 15°C. Pellets were resuspended in 10ml PBS and centrifuged at 1200rpm for 10 minutes at 4°C. After centrifugation, the cell pellets were resuspended in 5 ml PBS. A 5 μl aliquot of each sample was mixed with 95μl PBS and 100μl Trypan Blue for cell counting. The remaining cells were centrifuged at 1200rpm for 10 minutes at 4°C, resuspended in freezing media at 5x10^6^ cells/ml, aliquoted into 1ml cryotubes and placed into propanol filled freezing boxes overnight at -80°C. After one night the cells were transferred to liquid nitrogen for storage.
