# Supplementary material for: Short-term markers of DNA damage among roofers who work with hot asphalt
Source: Environ Health. 2016 Oct 20;15:99. doi: 10.1186/s12940-016-0182-4 (PMC5072307; doi:10.1186/s12940-016-0182-4)
Supplement: Additional file 3: Table S1. — Geometric means (and geometric standard deviations) of PAH measures in personal air samples (ng/m3) by study day and smoking status. Table S2. Geometric means (and geometric standard deviations) of urinary biomarkers (μg/g creatinine) in samples collected before and after work on both study days by smoking status. Table S3. Correlation between PAH exposure and biomarker data. Pearson correlation coefficients (and p-values) of log transformed measurements are presented for Monday (upper clear cells) and Thursday (lower grey shaded cells) post-shift samples. Urinary PAH metabolites and 8-OHdG are adjusted for urine creatinine. FLT = filter/particulate phase, XAD = adsorbent tube/gaseous phase, DERM = dermal wipe samples. (DOCX 36 kb) [file 12940_2016_182_MOESM3_ESM.docx]

**Additional file 3**

**Table S1** Geometric means (and geometric standard deviations) of PAH measures in personal air samples (ng/m^3^) by study day and smoking status

| **Airborne exposure measurements by study day and smoking status**  **(n=8 smokers, n=12 nonsmokers)** | | | | |
| --- | --- | --- | --- | --- |
|  | **Monday** | | **Thursday** | |
|  | **Nonsmokers** | **Smokers** | **Nonsmokers** | **Smokers** |
| **Naphthalene (ng/m^3^, XAD)** | 281.5 (2.0) | 354.2 (3.0) | 242.3 (3.0) | 572.5 (3.5) |
| **Pyrene (ng/m^3^, XAD)** | 1.7 (7.0) | 1.7 (9.0) | 1.3 (8.5) | 5.2 (6.2) |
| **Naphthalene (ng/m^3^, FLT)** | 0.534 (2.7) | 1.2 (2.4) | 0.839 (2.9) | 0.622 (2.8) |
| **Benzo(e)pyrene (ng/m^3^, FLT)** | 1.1 (7.1) | 3.5 (9.3) | 2.4 (8.7) | 10.2 (8.5) |

FLT: PAHs in particulate phase from filters; XAD: PAHs in gaseous phase from XAD adsorbent tubes

**Table S2** Geometric means (and geometric standard deviations) of urinary biomarkers (μg/g creatinine) in samples collected before and after work on both study days by smoking status

| **Urinary biomarkers (μg/g creatinine) before and after work by study day and smoking status**  **(n=8 smokers and n=12 nonsmokers)** | | | | | | | | |
| --- | --- | --- | --- | --- | --- | --- | --- | --- |
|  | **Monday** | | | | **Thursday** | | | |
|  | **Nonsmoker** | | **Smoker** | | **Nonsmoker** | | **Smoker** | |
| **Biomarker** | **Before** | **After** | **Before** | **After** | **Before** | **After** | **Before** | **After** |
| **1-OHNap** | 6,311 (4) | 11,160 (3) | 23,388* (4) | 40,135*,† (2) | 8,434 (2) | 11,849 (2) | 28,567* (2) | 25,336 (6) |
| **2-OHNap** | 54,176 (2) | 73,130 (3) | 58,105 (2) | 73,130 (2) | 44,356 (2) | 49,021 (2) | 97,734*,† (2) | 89,322* (2) |
| **1-OHPyr** | 953 (4) | 2,807 (3) | 578† (4) | 1737¥ (3) | 665 (7) | 1,588 (6) | 1,380  (3) | 1,939 (2) |
| **8-OHdG** | 2,515 (2) | 3,533 (1) | 1978† (2) | 4,492*,¥  (2) | 2,253 (1) | 3,905* (2) | 2,807  (2) | 3,828* (2) |

*p<0.05 or when compared to levels of non-smokers before work; †p<0.05 when compared to levels of non-smokers after work within same day; ¥ p<0.05 when compared to levels of smokers before work within same day.

**Table S3.** Correlation between PAH exposure and biomarker data. Pearson correlation coefficients (and p-values) of log transformed measurements are presented for Monday (upper clear cells) and Thursday (lower grey shaded cells) post-shift samples. Urinary PAH metabolites and 8-OHdG are adjusted for urine creatinine. FLT=filter/particulate phase, XAD=adsorbent tube/gaseous phase, DERM=dermal wipe samples.

| **Monday**  **Thursday** | **Naph (DERM)** | **Pyrene (DERM)** | **Pyrene**  **(XAD)** | **Naph**  **(XAD)** | **Naph**  **(FLT)** | **1-OHNap** | **2-OHNap** | **1-OHPyr** | **γH2ax** | **8-OHdG** |
| --- | --- | --- | --- | --- | --- | --- | --- | --- | --- | --- |
| **Naph (DERM)** | 1 | 0.545  (0.013) | -0.184  (0.436) | 0.399  (0.081) | -0.060  (0.809) | -0.024  (0.920) | 0.155  (0.514) | -0.083  (0.728) | 0.376  (0.102) | -0.019  (0.936) |
| **Pyrene (DERM)** | 0.334  (0.162) | 1 | 0.276  (0.238) | 0.649  (0.002) | -0.159  (0.502) | -0.240  (0.308) | 0.08  (0.750) | -0.10  (0.687) | -0.019  (0.938) | -0.120  (0.615) |
| **Pyrene**  **(XAD)** | 0.067  (0.782) | 0.006  (0.979) | 1 | 0.527  (0.017) | -0.068  (0.777) | -0.067  (0.780) | -0.265  (0.259) | 0.423  (0.063) | -0.290  (0.214) | 0.184  (0.438) |
| **Naph**  **(XAD)** | 0.154  (0.516) | 0.238  (0.326) | 0.488  (0.029) | 1 | -0.06  (0.798) | -0.09  (0.690) | -0.09  (0.694) | 0.068  (0.774) | -0.103  (0.665) | 0.160  (0.500) |
| **Naph**  **(FLT)** | -0.424  (0.063) | 0.241  (0.320) | -0.083  (0.723) | 0.02  (0.932) | 1 | 0.061  (0.799) | -0.01  (0.977) | -0.073  (0.759) | 0.192  (0.418) | -0.09  (0.712) |
| **1-OHNap** | 0.312  (0.181) | -0.192  (0.430) | 0.466  (0.038) | 0.177  (0.456) | 0.006  (0.981) | 1 | 0.622  (0.003) | 0.137  (0.563) | -0.050  (0.840) | 0.061  (0.798) |
| **2-OHNap** | 0.066  (0.783) | -0.258  (0.286) | 0.134  (0.573) | 0.03  (0.908) | -0.056  (0.813) | 0.517  (0.020) | 1 | 0.119  (0.616) | -0.246  (0.296) | -0.453  (0.050) |
| **1-OHPyr** | 0.551  (0.012) | 0.011  (0.964) | -0.146  (0.540) | -0.105  (0.660) | -0.323  (0.164) | 0.005  (0.984) | 0.209  (0.377) | 1 | -0.334  (0.150) | -0.243  (0.302) |
| **γH2ax** | 0.001  (0.996) | -0.322  (0.178) | -0.032  (0.895) | -0.282  (0.241) | 0.045  (0.856) | 0.576  (0.01) | 0.562  (0.012) | -0.054  (0.827) | 1 | 0.376  (0.103) |
| **8-OHdG** | -0.284  (0.239) | -0.286  (0.235) | -0.349  (0.143) | -0.158  (0.519) | 0.164  (0.502) | -0.039  (0.873) | 0.049  (0.840) | 0.097  (0.692) | 0.202  (0.406) | 1 |
